# Predictors of Unfavorable Outcomes in COVID-19-Related Sepsis: A Prospective Cohort Study

**DOI:** 10.3390/v17040455

**Published:** 2025-03-21

**Authors:** Diana-Maria Mateescu, Ioana Cotet, Cristina Guse, Catalin Prodan-Barbulescu, Norberth-Istvan Varga, Stela Iurciuc, Maria-Laura Craciun, Adrian-Cosmin Ilie, Alexandra Enache

**Affiliations:** 1 Doctoral School, Department of General Medicine, “Victor Babes” University of Medicine and Pharmacy, Eftimie Murgu Square 2, 300041 Timisoara, Romania; diana.mateescu@umft.ro (D.-M.M.); ioana.cotet@umft.ro (I.C.); cristina.marin@umft.ro (C.G.); catalin.prodan-barbulescu@umft.ro (C.P.-B.); norberth.varga@umft.ro (N.-I.V.); 2 Cardiology Department, “Victor Babes” University of Medicine and Pharmacy, Eftimie Murgu Square 2, 300041 Timisoara, Romania; iurciuc.stela@umft.ro (S.I.); laura.craciun@umft.ro (M.-L.C.); 3 Department III Functional Sciences, Division of Public Health and Management, “Victor Babes” University of Medicine and Pharmacy, Eftimie Murgu Square 2, 300041 Timisoara, Romania; 4 Discipline of Forensic Medicine, Bioethics, Deontology and Medical Law, Department of Neuroscience, “Victor Babes” University of Medicine and Pharmacy, Eftimie Murgu Square 2, 300041 Timisoara, Romania; enache.alexandra@umft.ro; 5 Ethics and Human Identification Research Center, Department of Neuroscience, Discipline of Forensic Medicine, Bioethics, Deontology and Medical Law, “Victor Babes” University of Medicine and Pharmacy, Eftimie Murgu Square 2, 300041 Timisoara, Romania

**Keywords:** COVID-19, severe COVID-19, sepsis, viral sepsis, COVID-19-related sepsis, sepsis biomarkers

## Abstract

Sepsis is a leading cause of mortality in critically ill patients, arising from a dysregulated immune response to infection. While traditionally associated with bacterial pathogens, severe COVID-19 can induce a sepsis-like syndrome, characterized by systemic inflammation, endothelial dysfunction, and coagulation abnormalities. This study aimed to assess the prognostic value of age, inflammatory markers, coagulation dysfunction, comorbidity burden, and lung involvement on computer tomography (CT) scans in predicting poor outcomes. We conducted a prospective cohort study including 163 patients diagnosed with COVID-19-related sepsis. Univariate and multivariable logistic regression analyses were performed to identify the independent predictors of unfavorable outcomes. Higher D-dimer (OR: 1.417, *p* = 0.020) and C-reactive protein (CRP) levels (OR: 1.010, *p* = 0.027) were independently associated with poor outcomes. A greater than 50% lung involvement on CT (OR: 1.774, *p* = 0.025) was also a significant predictor. The Charleson Comorbidity Index (CCI) showed a strong trend toward significance (*p* = 0.065), while age lost statistical significance after adjusting for comorbidities. Our findings suggest that D-dimers, CRP, and lung involvement on CT are key independent predictors of poor outcomes in COVID-19-related sepsis. These results emphasize the importance of inflammatory and coagulation markers, alongside comorbidity burden, in early risk assessment. Further prospective studies are warranted to refine predictive models for severe COVID-19 cases complicated by sepsis.

## 1. Introduction

Sepsis is one of the most lethal complications in critically ill patients, arising from a dysregulated host response to infection that leads to widespread inflammation, endothelial damage, and multi-organ failure [1,2]. Defined by the Sepsis-3 criteria, sepsis is characterized by life-threatening organ dysfunction due to a maladaptive immune response—a delicate balance where the immune system, instead of containing the infection, turns against the host [3]. Central to this process is the cytokine storm, an uncontrolled release of pro-inflammatory mediators such as interleukins, which drive systemic inflammation and microvascular dysfunction [4,5]. Several biomarkers play an important role in sepsis diagnosis and prognosis: C-reactive protein (CRP) and procalcitonin (PCT) rise in response to systemic inflammation, lactate (LAC) indicates tissue hypoxia and metabolic distress, and D-dimers signal the activation of coagulation and fibrinolysis, often linked to disseminated intravascular coagulation in severe sepsis [6,7,8,9,10]. Epidemiologically, sepsis accounts for nearly 20% of global deaths, and among critically ill COVID-19 patients, sepsis-related complications remain a leading cause of ICU admission and mortality [11].

The emergence of SARS-CoV-2 reshaped our understanding of viral sepsis. COVID-19 is far more than a respiratory infection—it is a multi-systemic disease, characterized by endothelial dysfunction, thrombo-inflammation, and immune dysregulation [12,13]. Early in the pandemic, severe cases were largely attributed to acute respiratory distress syndrome (ARDS) caused by direct viral cytopathy and diffuse alveolar damage [14,15]. However, as data accumulated, it became clear that COVID-19 can induce a sepsis-like syndrome, fueled by an overexuberant immune response, widespread endothelial injury, and dysregulated coagulation pathways [12,16]. The inflammatory response in severe COVID-19 mimics bacterial sepsis, with marked elevations in CRP, IL-6, and D-dimers—all markers now widely recognized as diagnostic indicators. Additionally, COVID-19-related vasculitis and microthrombosis further exacerbate end-organ damage, with the lung being the primary target [17]. Chest CT scans in severe COVID-19 patients often reveal extensive bilateral infiltrates, ground-glass opacities, and lung consolidation, correlating with disease severity and risk of mortality [18,19].

Beyond its pathophysiological complexity, COVID-19 disproportionately affects older adults and individuals with multiple comorbidities. Advanced age, hypertension, diabetes, cardiovascular disease, and chronic lung disease have all been consistently linked to worse clinical outcomes [20,21]. The Charlson Comorbidity Index (CCI) is one of the most widely used scoring systems to quantify comorbidity burden, and its predictive value has been demonstrated in numerous infectious disease cohorts [22]. However, the composite and intertwined roles of age, comorbidities, biomarkers, lung involvement, and lung failure, in determining COVID-19-related sepsis prognosis is yet to be clarified.

Given this background, our study aimed to identify the key clinical and biochemical predictors of unfavorable outcomes in patients with COVID-19-related sepsis. By evaluating inflammatory markers (CRP, LAC, and PCT), coagulation dysfunction (D-dimers), radiological severity (lung involvement on CT), and comorbidity burden (CCI), we sought to determine which factors most strongly predict poor prognosis in this unique patient population. Understanding these predictors is essential for early risk stratification, guiding treatment decisions, and improving clinical outcomes in severe COVID-19 cases complicated by sepsis.

## 2. Materials and Methods

### 2.1. Study Design and Population

This was an observational, prospective cohort study conducted at the “Victor Babes” Infectious Diseases and Pneumoftiziology Hospital, a tertiary, major referral center for Western and South-Western Romania, located in Timisoara. Data were collected prospectively, starting from 1 September 2022 to 31 December 2024. The study population consisted of adult patients (≥18 years of age) admitted to the hospital with confirmed COVID-19 infection and sepsis. Patients were eligible for inclusion if they met the following criteria: (1) confirmed COVID-19 infection via reverse transcription polymerase chain reaction (RT-PCR) assay; (2) diagnosis of sepsis at hospital admission, according to the Sepsis-3 criteria [3]; (3) age ≥ 18 years; and (4) provision of written informed consent by the patient or their next-of-kin. Patients were admitted either through direct transfer from other healthcare facilities or from their homes, typically presenting initially to the emergency department.

Patients were excluded from the study if the following applied: (1) they were unable or unwilling to provide informed consent (or if their next-of-kin could not provide consent); (2) they did not meet the Sepsis-3 criteria for sepsis at admission; (3) they met criteria for septic shock at admission, defined as requiring mechanical ventilation and/or vasopressor support; or (4) they had incomplete data (e.g., missing chest CT assessments of lung damage).

Ethical approval for this study was obtained from the Ethical Committee of the “Victor Babes” University of Medicine and Pharmacy of Timisoara, Romania, and from the Ethical Committee of the “Victor Babes” Infectious Disease and Pneumoftisiology Hospital (reference number: 70/01.09.2022, revised 2174/10.03.2023). This study adhered to the ethical principles outlined in the Declaration of Helsinki, including obtaining informed consent from all participants or their legal representatives.

### 2.2. Data Collection

Upon hospital admission to the infectious diseases ward, all patients with confirmed COVID-19 infection and suspected sepsis underwent a thorough clinical evaluation. Data were prospectively recorded for those patients meeting the inclusion criteria and enrolled in the study cohort. Demographic data (age, sex), COVID-19 vaccination status (unvaccinated, Pfizer, Johnson & Johnson), smoking, frequent alcohol consumption, and body mass index (BMI) were collected. Pre-existing comorbidities were thoroughly assessed, including hypertension, diabetes, stroke/dementia, coronary artery disease, cardiac insufficiency (NYHA class II-IV), chronic obstructive pulmonary disease (COPD), chronic hepatitis/cirrhosis, tuberculosis, and other relevant conditions (psychiatric, hormonal). The Charlson Comorbidity Index (CCI) was calculated. Clinical presentation included days from symptom onset to admission, baseline oxygen saturation, and oxygen supplementation requirements. Baseline chest computer tomography (CT) scans were performed, with lung involvement categorized as <25%, 25–50%, or >50%. Laboratory biomarkers included total leukocyte count, neutrophil-to-lymphocyte ratio (NLR), erythrocyte sedimentation rate (ESR), C-reactive protein (CRP), interleukin-6 (IL-6), procalcitonin (PCT), lactate (LAC), and D-dimers. In-hospital treatments (antivirals, antibiotics, corticosteroids) were recorded. The primary outcome was a composite unfavorable outcome, defined as a clinical evolution to one of the following events: (1) death during hospitalization, (2) septic shock, (3) mechanical ventilation, (4) vasopressors, or (5) Intensive Care Unit (ICU) transfer for any reason.

### 2.3. Statistical Analysis

Descriptive statistics were used to summarize patient characteristics. Continuous variables were presented as means and standard deviations (SDs). Normality was assessed using histograms and the Shapiro–Wilk test. Categorical variables were presented as frequencies and percentages. Univariate comparisons between patients with favorable and unfavorable outcomes were performed using the independent samples *t*-test for normally distributed continuous variables, the Mann–Whitney U test for non-normally distributed continuous variables, and the Chi-square test or Fisher’s exact test (as appropriate) for categorical variables. Multivariable logistic regression analysis was performed to identify the independent predictors of unfavorable outcomes. Odds ratios (ORs) with 95% confidence intervals (CIs) and *p*-values were reported. Model fit was assessed using the Hosmer–Lemeshow goodness-of-fit test. All statistical analyses were performed using SPSS Statistics version 26 (IBM Corp., Armonk, NY, USA). A *p*-value < 0.05 was considered statistically significant.

## 3. Results

### 3.1. General Characteristics of Study Population

This study included 163 patients admitted to an infectious diseases ward with a diagnosis of COVID-19-related sepsis, according to Sepsis-3 criteria. The cohort’s demographic, clinical, and treatment characteristics are summarized in Table 1. The mean age of the patients was 72.33 years (SD = 10.42), with a slight predominance of males (92 patients, 56.4%) compared to females (71 patients, 43.6%). As expected, almost all patients with COVID-19-related sepsis were unvaccinated against COVID-19 (159 patients, 97.5%). Only one patient (0.6%) had received the Pfizer vaccine, and three patients (1.8%) had received the Johnson & Johnson vaccine. The average time from symptom onset to hospital admission was 3.87 days (SD = 1.42). Fifty patients (30.7%) reported being current smokers, and 62 patients (38.0%) reported frequent (or daily) alcohol consumption. The mean body mass index (BMI) was 28.80 (SD = 5.06). BMI categorization revealed that 49 patients (30.1%) had a normal weight, 47 patients (28.8%) were overweight, 28 patients (17.2%) had class I obesity, 23 patients (14.1%) had class II obesity, and 16 patients (9.8%) had class III obesity.

Comorbidities were common in this cohort, reflecting the old age of patients with sepsis from COVID-19. The most frequent comorbidities were hypertension, present in 137 patients (84.0%), followed by diabetes, present in 59 patients (36.2%). Other frequent conditions were stroke, vascular or mixed dementia in 38 patients (23.3%), coronary artery disease in 35 patients (21.5%), and cardiac insufficiency in 28 patients (17.2%). Less frequent comorbidities included COPD (20 patients, 12.3%), chronic hepatitis/cirrhosis (14 patients, 8.6%), and a history of tuberculosis (8 patients, 4.9%). The mean Charles Comorbidity Index (CCI) in our cohort was 5.86 (SD = 2.72).

Regarding baseline disease severity upon admission, chest CT scans revealed varying degrees of lung involvement. The ‘lung involvement’ variable was recorded primarily as a continuous variable, but for this study, it was categorized into the following groups: <25% lung involvement (41 patients), 25–50% lung involvement (58 patients), and >50% lung involvement (64 patients). The mean oxygen saturation at baseline was 90.61% (SD = 6.35). A total of 111 patients (68.1%) required an oxygen flow rate greater than 15 L/min at baseline in order to keep the peripheral oxygen saturation above 93%. The mean SOFA score at admission was 6.65 (SD = 2.23), reflecting significant organ dysfunction in this cohort.

Baseline biomarker levels indicated significant systemic inflammation. The mean leukocyte count was 15.10 × 10^9^/L (SD = 5.47), the mean neutrophil-to-lymphocyte ratio (NLR) was 9.61 (SD = 2.97), the mean erythrocyte sedimentation rate (ESR) was 79.61 mm/hr (SD = 35.18), the mean C-reactive protein (CRP) was 126.15 mg/L (SD = 52.13), the mean IL-6 was 25.20 pg/mL (SD = 14.93), the mean procalcitonin was 2.19 ng/mL (SD = 1.13), the mean lactate was 2.02 mmol/L (SD = 0.63), and the mean D-dimer level was 1.16 μg/mL (SD = 0.89). Regarding treatment, 153 patients (93.9%) received the antiviral remdesivir, 142 patients (87.1%) received antibiotics, and 162 patients (99.4%) received corticosteroids (dexamethasone). In total, 44 patients experienced unfavorable outcomes, including 35 patients requiring mechanical ventilation, 27 needing vasopressors due to shock, and 39 were transferred to the ICU; only 2 patients in this group survived.

### 3.2. Univariate Analysis of Factors Associated with Unfavorable Outcomes

Table 2 presents the baseline characteristics of the 163 patients admitted with COVID-19-related sepsis, stratified by outcome (favorable vs. unfavorable). Univariate analyses were performed to identify the potential predictors of unfavorable outcomes. Firstly, we assessed the normality of continuous variables using the Shapiro–Wilk test in both outcome groups. Only BMI was normally distributed (*p* > 0.05) in both groups, so the independent *t*-test was used to verify whether there were significant differences regarding BMI between the two groups. All the other continuous variables (e.g., age, CCI, biomarkers, oxygen saturation, length of stay, etc.) were not normally distributed (*p* < 0.05), so the Mann–Whitney U test was chosen. CT severity was treated as a categorical variable, with three categories that reflected lung damage: <25%, 25–50%, and >50%. The Chi-square test was applied in this case. The other binary categorical variables (sex, vaccination, smoking, etc.) were tested using Fisher’s exact test.

Age was significantly higher in the unfavorable outcome group (79.45 years vs. 69.69 years, *p* < 0.001), indicating that older patients had a higher likelihood of experiencing an unfavorable outcome. The CCI score was significantly higher in the unfavorable outcome group (mean 6.87 vs. 5.06; *p* < 0.001), indicating that old age and an increasing comorbidity burden are associated with a greater risk of unfavorable outcomes. Similarly, the mean SOFA score was significantly higher in the unfavorable outcome group (8.21 vs. 5.86; *p* = 0.020). CT severity (expressed as percentage of lung involvement) was significantly associated with an unfavorable outcome (*p* = 0.033). Two baseline inflammatory markers were also significantly higher in the unfavorable outcome group: CRP (mean 140.58 mg/L vs. 120.83 mg/L; *p* = 0.018) and D-dimers (mean 1.48 μg/mL, vs. 1.04 μg/mL; *p* = 0.021).

While not reaching conventional statistical significance (*p* < 0.05), there was a higher proportion of patients with an oxygen flow >15 L/min at baseline (88.63% vs. 60.50%; *p* = 0.066). PCT also demonstrated higher plasma values in the unfavorable outcome group (mean 1.67 ng/mL vs. 2.76 ng/mL;), but this finding was close to reaching statistical significance (*p* = 0.074).

No statistically significant differences were observed between the two outcome groups in terms of sex, smoking status, alcohol consumption, BMI, baseline oxygen saturation, and the baseline plasma levels of total leukocyte count, NLR, ESR, and LAC (all *p* > 0.05). Regarding vaccination status, the vast majority of patients (97.5%) were unvaccinated, limiting the statistical power to detect differences between groups. There was also no statistically significant association of treatment with antivirals, antibiotics, or corticosteroids with outcome (all *p*-values > 0.05).

### 3.3. Multivariable Analysis of Predictors of Unfavorable Outcomes

A binary logistic regression analysis was conducted to identify independent predictors of unfavorable outcomes in patients with COVID-19-related sepsis. Based on the results of the univariate analysis, age, CCI, and baseline CRP and D-dimer plasma values were included as continuous variables in the final model. The SOFA score was not included in the multivariable model, despite its univariate significance (*p* = 0.020), as its composite nature overlaps with CCI and D-dimers, and this study focuses on specific predictors beyond baseline severity. Lung involvement was used as a categorical variable, as described previously. Given the strong correlation between age and CCI, a multicollinearity test was performed before including both variables, and the linear regression analysis confirmed that collinearity was not a major concern (VIF = 2.919 for both variables). The Hosmer–Lemeshow test yielded a *p*-value of 0.058, indicating good model fit (*p* > 0.05), with acceptable calibration.

The analysis revealed that CRP and D-dimers were significant independent predictors of an unfavorable outcome. Each 1 mg/L increase in CRP was associated with a 1.01-fold increase in the odds of an unfavorable outcome (OR: 1.010; 95% CI: 1.001–1.018, *p* = 0.027), while higher D-dimer levels were also strongly associated with increased risk (OR: 1.417, 95% CI: 1.056–1.903, *p* = 0.020). A greater than 50% lung involvement, as observed on the CT scan at hospital admission, was also an independent predictor of poor outcome (OR: 1.774; 95% CI: 1.13–3.856; *p* = 0.025). CCI showed a trend toward statistical significance (OR: 1.579, 95% CI: 0.972–2.564, *p* = 0.060), suggesting that comorbidity burden may play a role in clinical outcomes. Age was not statistically significant after adjusting for CCI (OR: 1.080, 95% CI: 0.994–1.174, *p* = 0.071). The results of the multivariable logistic regression analysis are presented in Table 3.

## 4. Discussion

This study aimed to identify clinical and biochemical predictors of unfavorable outcomes in patients with COVID-19-related sepsis, using both univariate and multivariable analyses. Given the high mortality associated with sepsis in COVID-19 patients, the early identification of risk factors is essential for guiding treatment decisions and improving prognosis.

In the univariate analysis, several variables were significantly associated with unfavorable outcomes. Older age, a higher Charlson Comorbidity Index, greater lung involvement on CT scans, elevated CRP, and increased D-dimer levels were all significantly more prevalent in patients with poor outcomes. Additionally, a trend toward significance was observed for oxygen flow >15 L/min and procalcitonin levels, though these did not reach conventional statistical significance. Multivariable logistic regression identified CRP and D-dimers as independent predictors of unfavorable outcomes. Each 1 mg/L increase in CRP was associated with a 1% increase in the odds of an unfavorable outcome (*p* = 0.027), while each 1 μg/mL increase in D-dimers was associated with a 41.7% increase in the odds of poor prognosis (OR = 1.417, 95% CI: 1.056–1.903, *p* = 0.020). These findings reinforce the role of coagulation disturbances in COVID-19-related sepsis and highlight the prognostic value of inflammatory and thrombotic biomarkers. Additionally, patients with a greater than 50% lung involvement on CT scans had a 77% higher risk of an unfavorable outcome compared to those with <25% of lung involvement (OR = 1.774, *p* = 0.025), reinforcing the role of severe pulmonary damage in predicting prognosis. While age and CCI did not reach conventional statistical significance, both showed a trend toward significance (*p* = 0.071 and *p* = 0.065, respectively), suggesting that comorbidity burden and advanced age may still contribute to prognosis in COVID-19-related sepsis. These findings emphasize the multifactorial nature of disease severity, where inflammatory markers (CRP), coagulation dysfunction (D-dimers), and lung damage (CT severity score) play key roles in determining patient outcomes. Beyond these predictors, our univariate analysis revealed a significant difference in SOFA scores between outcome groups (8.21 vs. 5.86, *p* = 0.020), highlighting the role of baseline organ dysfunction in driving unfavorable outcomes. However, we opted not to include SOFA in the multivariable model, as its composite nature overlaps with CCI and D-dimers, potentially masking the specific contributions of our targeted predictors. This choice kept our focus on distinct, actionable markers rather than broad severity indices.

Notably, 97.5% of our cohort was unvaccinated, a striking feature that reflects the specific population presenting to our clinic during this phase of the pandemic, many of whom delayed seeking care until sepsis had set in. This near-uniform lack of vaccination precluded meaningful statistical comparison between vaccinated and unvaccinated patients, though it underscores the vulnerability of this group to severe outcomes. As such, our findings must be interpreted within this context.

The findings of this study align with a growing body of evidence, highlighting the key clinical and biochemical predictors of severe COVID-19 outcomes. Our results support previous research indicating that higher CCI scores are associated with an increased risk of severe outcomes in COVID-19. In our study, while CCI did not reach full statistical significance in the multivariable model (*p* = 0.065), it showed a strong trend toward predicting unfavorable outcomes, suggesting that comorbidity burden plays a key role in disease severity. Similar findings were reported by Comoglu et al. (2022), who found that each point increase in CCI corresponded to a 1.53-fold increase in mortality risk [23]. Likewise, Kim et al. (2021) demonstrated that the Age-Adjusted CCI was the most reliable predictor of severe COVID-19 outcomes among hospitalized patients, outperforming other commonly used risk stratification tools [24]. Türker et al. (2022) also highlighted the value of CCI in early mortality prediction, particularly when combined with inflammatory markers [25]. Taken together, these studies reinforce the notion that comorbidity burden—rather than age alone—may be a more meaningful determinant of prognosis in older adults with COVID-19.

Our study identified elevated D-dimer levels as an independent predictor of unfavorable outcomes (OR = 1.417, *p* = 0.020), a finding that aligns with multiple prior investigations. Yao et al. (2020) found that D-dimer levels above 2.0 μg/mL were associated with a 6-fold increase in mortality, reinforcing its role as a critical biomarker for thrombotic complications in COVID-19 [26]. Huang et al. (2020) confirmed this association in a meta-analysis, reporting that D-dimer was one of the strongest biochemical predictors of severe disease and mortality [27]. Additionally, Marin et al. (2020) observed that D-dimer levels >1.5 μg/mL were highly predictive of ICU admission and the need for mechanical ventilation [28].

The significant association between CRP levels and poor outcomes in our study (OR = 1.010, *p* = 0.027) is consistent with previous research emphasizing the role of inflammation in COVID-19 progression. Ji et al. (2020) found that CRP levels above 100 mg/L were strongly predictive of ICU admission, suggesting its role as a biomarker for early risk stratification [29]. Similarly, a meta-analysis by Elshazli et al. (2020) (6320 patients) confirmed that elevated CRP was strongly correlated with disease severity and mortality [30]. Huang et al. (2020) also included CRP in their systematic review of inflammatory markers in COVID-19, reinforcing its prognostic value [27]. Our results corroborate these findings, further demonstrating that elevated systemic inflammation is a central driver of severe disease progression in COVID-19-related sepsis.

We found that patients with >50% lung involvement had a significantly higher risk of poor outcomes (OR = 1.774, *p* = 0.025), a finding that aligns with prior research, emphasizing the prognostic value of chest CT severity scoring. Francone et al. (2020) reported that patients with a >50% lung involvement had significantly higher ICU admission rates and mortality risks [31]. Moreover, Alilou et al. (2023) highlighted that radiological severity scores are predictive not only of acute outcomes but also of long-term pulmonary sequelae in COVID-19 survivors [32].

While most of our findings align with prior studies, some differences emerged. Age did not remain a significant predictor in our multivariable analysis (*p* = 0.071), which contrasts with several large-scale studies. Incerti et al. (2021) found that age was one of the strongest independent predictors of mortality in hospitalized COVID-19 patients [33], while Jaskolowska et al. (2023) identified age ≥ 75 years as a critical threshold for increased mortality [34]. Shi et al. (2021), in a meta-analysis, reinforced that age remains a key determinant of severe disease [35]. The literature consistently shows that older patients face higher mortality, particularly with elevated D-dimers and CRP [27]; yet in our cohort, age lost statistical significance in multivariable analysis, likely overshadowed by the heavy comorbidity burden captured by the CCI. Similarly, while males are reported to exhibit higher inflammatory markers and worse outcomes due to a more robust inflammatory response [36], we observed no significant gender difference (*p* = 0.812), despite a slight male predominance (56%). This may reflect the specific dynamics of COVID-19-related sepsis in our cohort, where the sepsis syndrome, marked by overwhelming inflammation and coagulation dysfunction, might level the playing field across demographics, leaving biomarkers and lung damage as the louder signals in this particular fight.

Our reliance on Sepsis-3 criteria to define COVID-19-related sepsis, while effective for capturing severe cases, raises questions about alternative definitions. Sepsis-2 criteria [37] might better discriminate suspected sepsis or mortality in this context, potentially identifying a broader patient spectrum [38]. Adopting Sepsis-2, for instance, could have included milder cases, possibly altering the weight of predictors like age or CCI—though our focus on confirmed sepsis likely sharpened the role of biomarkers and lung involvement in this cohort.

### Limitations and Future Research Directions

Our study has some noteworthy limitations. Firstly, as a single-center, observational cohort study, causality and generalizability are limited. While our results align with the previous literature, conducting a multi-center study with a larger and more diverse patient population could provide more robust evidence and improve external validity. Secondly, although we included multiple clinical and biochemical variables in our multivariable regression model, it is possible that unmeasured confounders influenced the outcomes. Factors such as specific viral variants, prior outpatient treatments, and socioeconomic status were not accounted for in our analysis, and these may play a role in determining prognosis in COVID-19-related sepsis. Third, the timing of biomarker measurements was based on admission values, which might not fully capture the dynamic changes in inflammatory and coagulation markers throughout hospitalization. Serial measurements of CRP, D-dimers, and other biomarkers could provide a more comprehensive understanding of their predictive value over the course of illness. Blood gasses, renal and liver markers, underpinned our Sepsis-3 diagnosis via SOFA scores, reported here as baseline severity measures rather than individual predictors—though their detailed profiling could have further refined our risk assessment. Finally, our study focused on in-hospital outcomes without assessing long-term prognosis or post-discharge complications. Given the increasing recognition of post-acute sequelae of COVID-19 (PASC), future research should explore how early biomarkers correlate with long-term functional outcomes in COVID-19 sepsis survivors.

While our findings highlight D-dimers, CRP, and lung involvement as key predictors of poor outcomes in COVID-19-related sepsis, several factors warrant further consideration. Age and comorbidity burden, as captured by the CCI, showed only a trend toward significance (*p* = 0.071 and *p* = 0.060, respectively), yet their clinical relevance likely persists—older patients with heavier comorbidity loads often walked a tighter rope in our wards. Treatment protocols, though standardized, varied slightly in timing and combination across patients, potentially nudging outcomes in ways our analysis could not fully tease out. The severity of illness at admission, reflected in baseline oxygen needs and CT findings, also likely shaped prognosis, with those arriving sicker facing steeper odds, regardless of biomarkers. All these confounding variables remind us that patients with severe COVID-19 and sepsis represent a complex medical puzzle, and their interplay limits the robustness of our predictors in capturing the full scope of this disease.

## 5. Conclusions

In this observational cohort study of patients with COVID-19-related sepsis, we identified D-dimers, CRP, and lung involvement on CT as independent predictors of unfavorable outcomes. While age and CCI showed a strong trend toward significance, our findings suggest that comorbidity burden and systemic inflammation may play a more critical role than age alone in determining prognosis. These results reinforce the importance of early risk stratification using inflammatory and coagulation biomarkers, alongside radiological assessment. Given the single-center, observational cohort nature of this study, further multi-center research is needed to validate these findings and refine predictive models for severe COVID-19 outcomes.

## Figures and Tables

**Table 1:** Baseline characteristics of patients admitted with COVID-19-associated sepsis. CCI = Charleson Comorbidity Index; BMI = body mass index; COPD = chronic obstructive pulmonary disease; NLR = neutrophil-to-leukocyte ratio; ESR = erythrocyte sedimentation rate; CRP = C-reactive protein; IL-6 = interleukin-6; PCT = procalcitonin; LAC = lactate.

Characteristic	Overall (*n* = 163)
Age (years), Mean (SD)	72.33 (10.42)
Male Sex, *n* (%)	92 (56.4)
Vaccination Status	
Unvaccinated, *n* (%)	159 (97.5)
Vaccinated—Pfizer, *n* (%)	1 (0.6)
Vaccinated—Johnson&Johnson, *n* (%)	3 (1.8)
Days to Admission	3.87 (1.4)
Smoking, *n* (%)	50 (30.7)
Frequent Alcohol Consumption, *n* (%)	62 (38.0)
BMI, Mean (SD)	28.80 (5.06)
Normal Weight, *n* (%)	49 (30.1)
Overweight, *n* (%)	47 (28.8)
Obesity Class I, *n* (%)	28 (17.2)
Obesity Class II, *n* (%)	23 (14.1)
Obesity Class III, *n* (%)	16 (9.8)
Comorbidities	
CCI Score, Mean (SD)	5.01 (1.6)
Hypertension, *n* (%)	137 (84.0)
Diabetes, *n* (%)	59 (36.2)
Stroke/Dementia, *n* (%)	38 (23.3)
Coronary Artery Disease, *n* (%)	35 (21.5)
Cardiac Insufficiency, *n* (%)	28 (17.2)
COPD, *n* (%)	20 (12.3)
Chronic Hepatitis/Cirrhosis, *n* (%)	14 (8.6)
History of Tuberculosis, *n* (%)	8 (4.9)
CT Severity Score	
<25% Lung Involvement, *n* (%)	41 (25.2)
25–50% Lung Involvement, *n* (%)	58 (35.6)
>50% Lung Involvement, *n* (%)	64 (39.3)
Oxygen Saturation at Baseline, Mean (SD)	90.61 (6.35)
Oxygen Flow > 15 L/min at Baseline, *n* (%)	111 (68.1)
SOFA Score, Mean (SD)	6.65 (2.3)
Baseline Biomarkers, Mean (SD)	
Leukocytes (×10^9^/L)	15.10 (5.47)
NLR	9.61 (2.97)
ESR (mm/hr)	79.61 (35.18)
CRP (mg/L)	126.15 (52.13)
IL-6 (pg/mL)	30.68 (14.93)
PCT (ng/mL)	2.19 (1.13)
LAC (mmol/L)	2.02 (0.63)
D-Dimers	1.16 (0.89)
Treatment	
Antiviral (Remdesivir), *n* (%)	153 (93.9)
Antibiotics, *n* (%)	142 (87.1)
Corticosteroids, *n* (%)	162 (99.4)
Length of Stay (days), Mean (SD)	13.91 (7.87)

**Table 2:** Baseline characteristics of study population, stratified by outcome. CCI = Charleson Comorbidity Index; COPD = chronic obstructive pulmonary disease; NLR = neutrophil-to-leukocyte ratio; ESR = erythrocyte sedimentation rate; CRP = C-reactive protein; IL-6 = interleukin-6; PCT = procalcitonin; LAC = lactate.

Characteristic	Favorable Outcome (*n* = 119)	Unfavorable Outcome (*n* = 44)	*p*-Value
Age (years), Mean (SD)	69.69 (9.26)	79.45 (10.13)	<0.001
Male Sex, *n* (%)	66 (55.46)	26 (59.09)	0.812
Vaccination Status			0.381
Unvaccinated, *n* (%)	115 (96.63)	44 (100)	
Vaccinated—Pfizer, *n* (%)	1 (0.84)	0 (0)	
Vaccinated—Johnson&Johnson, *n* (%)	3 (2.52)	0 (0)	
Days to Admission	3.43 (1.22)	4.58 (1.94)	0.449
Smoking, *n* (%)	36 (30.25)	14 (31.81)	0.617
Frequent Alcohol Consumption, *n* (%)	40 (33.61)	22 (50)	0.104
BMI, Mean (SD)	28.11 (5.1)	29.42 (5.01)	0.502
CCI, Mean (SD)	4.57 (1.31)	6.20 (1.69)	<0.001
CT Severity, *n* (%)			0.033
<25% Lung Involvement	33 (27.73)	8 (18.18)	
25–50% Lung Involvement	40 (33.61)	18 (40.90)	
>50% Lung Involvement	37 (31.09)	27 (61.36)	
Baseline Oxygen Saturation, Mean (SD)	91.27 (4.83)	88.36 (7.92)	0.188
Oxygen Flow > 15 L/min at Baseline, *n* (%)	72 (60.50)	39 (88.63)	0.066
SOFA Score, Mean (SD)	5.86 (2.14)	8.21 (2.67)	0.020
Baseline Biomarkers, Mean (SD)			
Leukocytes (×10^9^/L)	14.93 (4.98)	15.58 (6.65)	0.383
NLR	8.96 (3.61)	10.11 (4.84)	0.092
ESR (mm/hr)	77.02 (35.41)	86.57 (41.46)	0.145
CRP (mg/L)	120.83 (48.69)	140.58 (86.66)	0.018
IL-6 (pg/mL)	25.35 (39.61)	51.39 (80.21)	0.849
PCT (ng/mL)	1.67 (1.39)	2.76 (3.15)	0.074
LAC (mmol/L)	1.96 (0.56)	2.2 (0.8)	0.110
D-Dimers	1.04 (0.87)	1.48 (1.04)	0.021
Treatment			
Antiviral (Remdesivir), *n* (%)	110 (92.43)	43 (97.72)	0.284
Antibiotics, *n* (%)	98 (82.35)	44 (100)	0.142
Corticosteroids, *n* (%)	119 (100)	43 (97.72)	0.627
Length of Stay (days), Mean (SD)	13.92 (8.23)	13.86 (6.92)	0.673

**Table 3:** Multivariable logistic regression model for predictors of unfavorable outcomes. CCI = Charleson Comorbidity Index; CRP = C-reactive protein.

Variable	OR	95% CI	*p*-Value
Age	1.080	0.994–1.174	0.071
CCI	1.579	0.0972–2.564	0.060
D-dimers	1.417	1.056–1.903	0.020
Lung Involvement	1.774	1.138–3.856	0.025
CRP	1.010	1.010–1.018	0.027

## Data Availability

The original contributions presented in the study are included in the article, and further inquiries can be directed to the corresponding author.

## Abbreviations

The following abbreviations are used in this manuscript:

CCI	Charleson Comorbidity Index
BMI	Body Mass Index
COPD	Chronic Obstructive Pulmonary Disease
NLR	Neutrophil-to-Lymphocyte Ratio
ESR	Erythrocyte Sedimentation Rate
CRP	C-Reactive Protein
IL-6	Interleukin 6
PCT	Procalcitonin
LAC	Lactate
CT	Computer Tomography
